# A neonate with Klippel–Trénaunay syndrome: a case report 

**DOI:** 10.1186/s13256-021-03029-4

**Published:** 2021-09-07

**Authors:** Franck Katembo Sikakulya, Walufu Ivan Egesa, Sonye Magugu Kiyaka, Philip Anyama

**Affiliations:** 1 Department of Surgery, Faculty of Clinical Medicine and Dentistry, Kampala International University Western Campus, Ishaka-Bushenyi, Uganda; 2 Faculty of Medicine, Université Catholique du Graben, Butembo, Democratic Republic of the Congo; 3 Department of Paediatrics and Child Health, Faculty of Clinical Medicine and Dentistry, Kampala International University, Bushenyi, Uganda; 4 Department of Surgery, Jinja Regional Referral Hospital, Jinja, Uganda

**Keywords:** Port wine stain, Hypertrophy, Vascular malformation, Klippel–Trénaunay syndrome

## Abstract

**Background:**

Klippel–Trénaunay syndrome is a rare congenital capillary–lymphatic–venous condition characterized by the clinical triad of capillary malformations (port wine stains), varicose veins with or without venous malformations, and bony and/or soft-tissue hypertrophy. It has a very low incidence of about 1:100,000.

**Case presentation:**

We report the case of 21-day-old neonate Black African female (born in Uganda) with Klippel–Trénaunay syndrome who presented with macrodactyly and ectrodactyly on the left foot, as well as numerous port wine stains on the left thoracoabdominal region and anteroposterior left lower limb. Color Doppler ultrasound examination of the left lower limb and abdomen revealed varicose veins without signs of arteriovenous fistula.

**Conclusion:**

The report presents the case of a neonate with a rare congenital vascular disorder type Klippel–Trénaunay syndrome.

## Introduction

Over a hundred years ago, French physicians Klippel and Trénaunay described for the first time a rare congenital disorder named Klippel–Trénaunay syndrome (KTS) [1] with a very low incidence of about 1:100,000 [2].

KTS is a capillary–lymphatic–venous malformation associated with soft-tissue and skeletal hypertrophy and is clinically recognized by a triad of capillary malformations (port wine stain), atypical venous malformations, and bony and/or soft-tissue hypertrophy; presence of any two of these features will confirm the diagnosis [3].

The syndrome is usually diagnosed at birth, but it can be found in older children and adults if not diagnosed in time. Extremities, particularly the lower extremities, are affected by vascular abnormalities [4]. Most cases of KTS are sporadic, but little has been published about family members suggesting an inherited disorder [2, 5].

We present the case of a neonate who was admitted with features of Klippel–Trénaunay syndrome.

## Case presentation

A 21-day-old neonate Black African female was delivered by normal spontaneous vaginal delivery at 39 weeks gestation with no complications. She was born to a 19-year-old healthy G1 + P1 + 0 mother who attended four antenatal-care visits. The mother’s pregnancy progressed without any complication or notable environmental exposures. No ultrasound scan was done during the pregnancy period. There was no family or parental history of inherited vascular disorder, and the parents were not blood relatives.

**Fig. 1:**
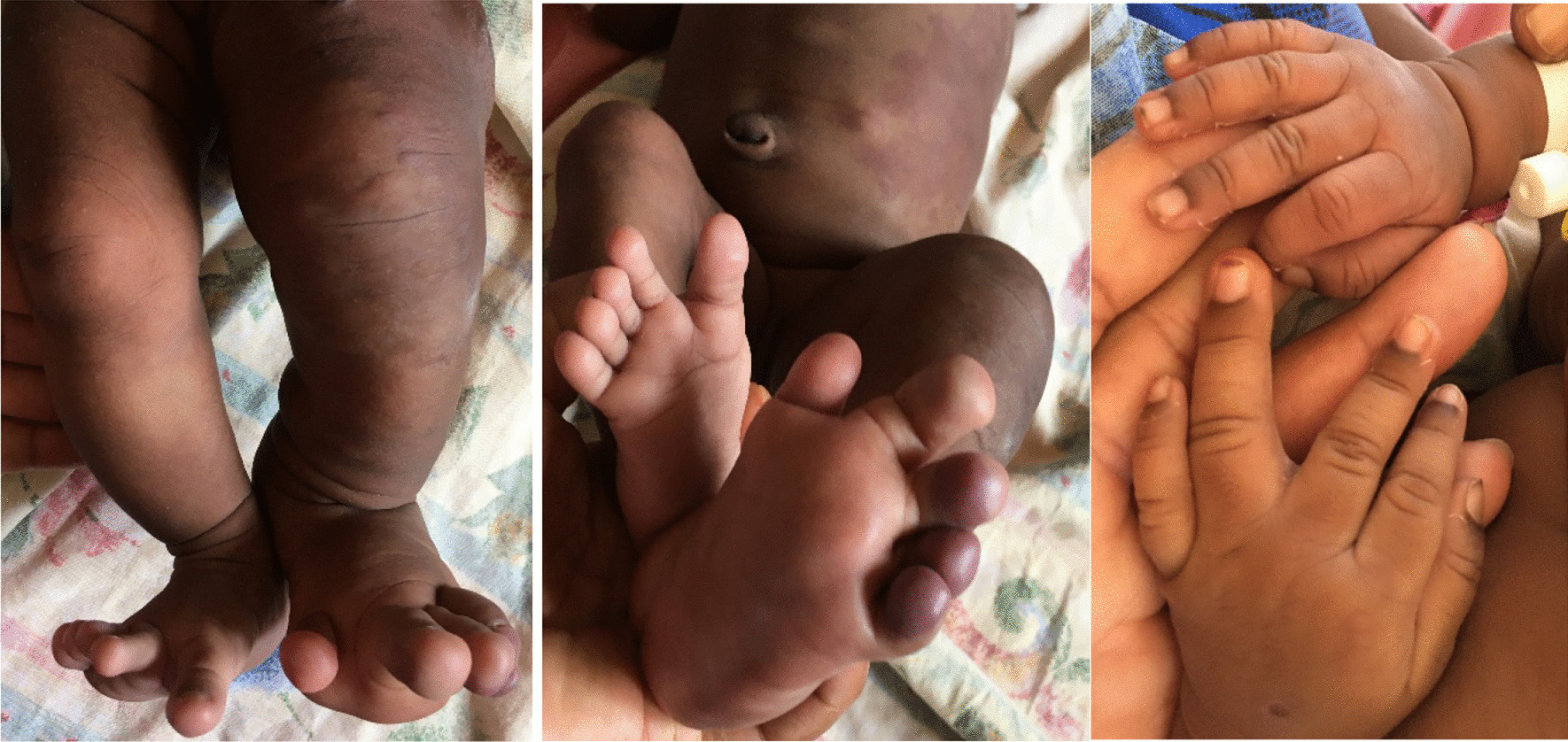
Enlargement of the left upper and lower extremity with macrodactyly and with ectrodactyly on the feet

**Fig. 2:**
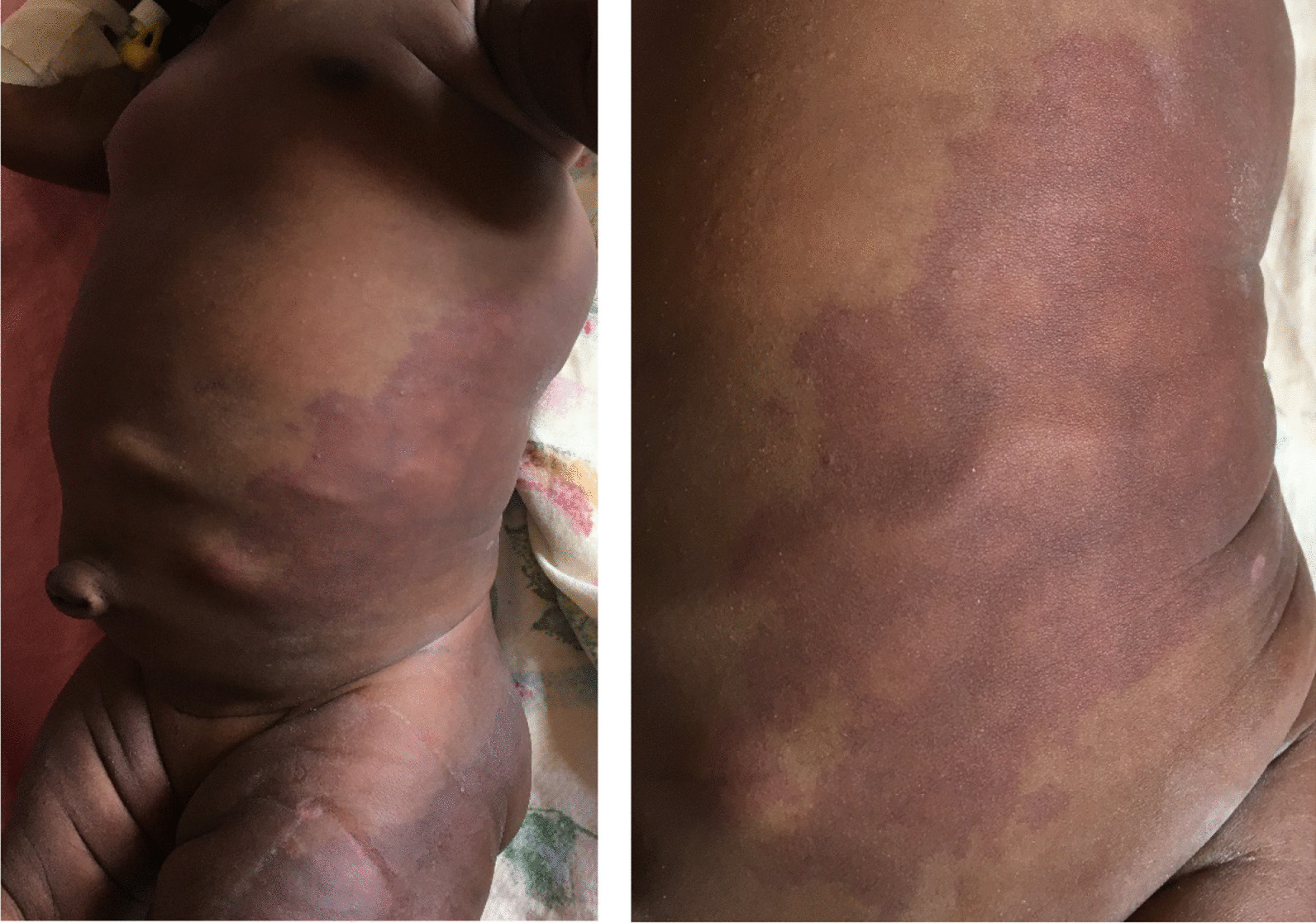
Thoracoabdominal port wine stains plus varicose veins on the abdomen

The neonate was delivered normally, weighed 3000 g, and was not resuscitated. On examination, the neonate had obvious enlargement of the left lower and left upper extremities (macrodactyly and ectrodactyly on the left foot) (Fig. 1) with numerous port wine stains on the left thoracoabdominal region and anteroposterior left lower limb (Fig. 2). Vascular lesions were seen on the abdomen (Fig. 2), heart sounds were normal with no murmur, and there were no respiratory or intraabdominal abnormalities. Neurological examination was intact. These features were compatible with a diagnosis of Klippel–Trénaunay syndrome. Full blood count reported thrombocytosis with a slight elevation of prothrombin time (16 seconds, reference [11–15]). We performed a color Doppler ultrasound examination of the left lower limb and abdomen, which showed varicose veins and no signs of arteriovenous fistula, confirming the clinical diagnosis of KTS. Nonavailability of equipment and financial constraint were the cause of not doing magnetic resonance imagery to support the Doppler ultrasound.

Patient was reviewed by orthopedic, ophthalmology, and ear, nose, throat (ENT) teams with no recommended intervention at that time.

A definitive diagnosis with genetic testing and extensive family history and examination was not possible due to financial constrains. The patient was discharged in good condition at 26 days of age. A continuous appointment was established for follow-up.

## Discussion

We have presented the case of a 21-day-old female with a rare vascular disorder type KTS. The neonate presented with macrodactyly and ectrodactyly on the left foot, numerous port wine stains on the left thoracoabdominal region and anteroposterior left lower limb without bleeding disorder. A color Doppler ultrasound examination revealed varicose veins without signs of arteriovenous fistula. The presence of ectrodactyly on the hand has been reported once in a live birth case [6], but presentation on the foot has not yet been presented. The 21-day-old female presented with ectrodactyly on the feet.

KTS is a rare congenital capillary–lymphatic–venous abnormality that usually involves a single lower extremity and consists of the triad of vascular malformations (port wine stain), atypical venous malformations, and bony and/or soft-tissue hypertrophy [3].

Its prevalence is low or underreported with a very low incidence of about 1:100,000. It has no predilection for gender, race, or geographical area and occurs sporadically [2].

Although the etiology of KTS is still unknown, damage to the sympathetic nervous system resulting in dilatation and persistence of microscopic arteriovenous anastomoses *in utero* is hypothesized to be the leading cause [7]. A Doppler ultrasound scan during pregnancy should be performed to diagnose the hypertrophic limb and to assess the underlying cause of any cystic lesion.

KTS has been shown to belong to a spectrum of segmental overgrowth diseases caused by mutations in the PIK3CA gene, which differentiates it from Parkes–Weber syndrome, which is caused by mutations of the RASA1 gene [8, 9]

Most patients with KTS will present with the classic triad [3], but some clinical variations can be seen with age (childhood and adulthood) [4]; however, these are far less common.

Few numbers or absence of lymphatic channels are leading causes of lymphedema that can be documented using ultrasound (with or without Doppler), magnetic resonance imaging (MRI), and vascular studies (arteriography, venography, and/or lymphography) [10, 11]. This patient presented with lymphedema on the back, and left lower limb (thigh to foot). The lymphatic anomalies can also occur in the pelvis, bladder, lower gastrointestinal tract, and spleen [10]. In the current case report, only Doppler ultrasound scan was done due to its affordability and availability compared with other investigations [11].

Extremities are mostly affected, most often unilaterally (85%), sometimes bilaterally (12.5%), and only rarely crossed-bilaterally (2.5%) [12]. The case presented in this report had left upper and lower limbs affected with macrodactyly and ectrodactyly on the left foot. Various other limb anomalies including camptodactyly, syndactyly, clinodactyly, and congenital hip dislocation have been reported in association with KTS [10].

Differential diagnosis for KTS should consider Prader–Willi syndrome (PWS), Proteus syndrome, Maffucci syndrome, neurofibromatosis type I, Sturge–Weber syndrome, and Beckwith–Wiedemann syndrome [10, 13, 14].

The presence of arteriovenous fistula in PWS is the only difference with KTS, and both syndromes are generally confirmed with Doppler ultrasound and magnetic resonance angiography [10, 13].

It was reported that KTS can be associated with deep venous thrombosis, bleeding, pulmonary embolism, stasis dermatitis, cellulitis, and limb enlargement, which can lead to amputation [15]. Currently, there is no definitive treatment of KTS approved; however, a multidisciplinary management should be focused on reducing symptoms and complications associated to the disease. For varicose veins, compressive shocks were reported to be used. Surgical and orthopedic management can be applied too for the extremity asymmetry and spine deformities such scoliosis. Port wine stains are treated with pulsed dye laser therapy [16].

## Conclusion

We presented the case of a neonate with a rare congenital vascular disorder type KTS who presented with macrodactyly and ectrodactyly on the left foot, as well as numerous port wine stains. This case serves as review of clinical features and etiopathology of KTS and also highlights the importance of a multidisciplinary management team and follow-up, which can help to avoid the occurrence of complications that have an impact on the prognosis of the patient.

## Data Availability

All data generated or analyzed during this study are included in this published article
